# *In silico* prediction and characterisation of secondary metabolite clusters in the plant pathogenic fungus *Verticillium dahliae*

**DOI:** 10.1093/femsle/fnz081

**Published:** 2019-04-20

**Authors:** Xiaoqian Shi-Kunne, Roger de Pedro Jové, Jasper R L Depotter, Malaika K Ebert, Michael F Seidl, Bart P H J Thomma

**Affiliations:** 1 Laboratory of Phytopathology, Wageningen University & Research, Droevendaalsesteeg 1, 6708 PB Wageningen, The Netherlands; 2 Department of Crops and Agronomy, National Institute of Agricultural Botany, Huntingdon Road, CB3 0LE Cambridge, United Kingdom

**Keywords:** Verticillium, pathogen, natural product, genomics, fungi

## Abstract

Fungi are renowned producers of natural compounds, also known as secondary metabolites (SMs) that display a wide array of biological activities. Typically, the genes that are involved in the biosynthesis of SMs are located in close proximity to each other in so-called secondary metabolite clusters. Many plant-pathogenic fungi secrete SMs during infection in order to promote disease establishment, for instance as cytocoxic compounds. *Verticillium dahliae* is a notorious plant pathogen that can infect over 200 host plants worldwide. However, the SM repertoire of this vascular pathogen remains mostly uncharted. To unravel the potential of *V. dahliae* to produce SMs, we performed *in silico* predictions and in-depth analyses of its secondary metabolite clusters. Using distinctive traits of gene clusters and the conserved signatures of core genes 25 potential SM gene clusters were identified. Subsequently, phylogenetic and comparative genomics analyses were performed, revealing that two putative siderophores, ferricrocin and TAFC, DHN-melanin and fujikurin may belong to the SM repertoire of *V. dahliae*.

## Teaser

In total, 25 potential SMCs were identified in the genome of the plant pathogenic fungus *Verticillium dahliae*, including loci that can be implicated in DHN-melanin, ferricrocin, triacetyl fusarinine and fujikurin production.

## INTRODUCTION

Filamentous fungi are known for their ability to produce a vast array of distinct chemical compounds that are also known as secondary metabolites (SMs) (Keller, Turner and Bennett 2005). In contrast to primary metabolites, SMs are often considered as non-essential for fungal growth, development or reproduction. However, SMs can be crucial for long-term survival in competitive fungal niches (Fox and Howlett 2008; Ponts 2015; Derntl *et al*. 2017). SMs produced by plant pathogenic fungi are of particular interest as they may contribute to virulence, leading to crop losses and threatening food security (Ponts 2015; Pusztahelyi, Holb and Pócsi 2015). For example, T-toxin from *Cochliobolus heterostrophus*, a maize pathogen that caused the worst epidemic in U.S. agricultural history, has been reported to be a crucial pathogenicity factor (Inderbitzin, Asvarak and Turgeon 2010).

Fungal SMs are classified into four main groups based on core enzymes and precursors involved in their biosynthesis: polyketides, non-ribosomal peptides, terpenes and indole alkaloids (Keller, Turner and Bennett 2005). Production of the chemical scaffold of each class requires core enzymes named polyketides synthases (PKSs), non-ribosomal peptide synthetases (NRPSs), terpene cyclases and dimethylallyl tryptophan synthases, respectively. Additionally, hybrid enzymes such as PKS-NRPSs have been identified as builders of structurally complex molecules with combined properties (Boettger and Hertweck 2013). PKSs and NRPSs are the most abundant and are extensively studied in fungi (Cox 2007). PKSs can be further divided into three different types (I, II and III), of which type I PKSs and type III PKSs are found in fungi. Type I PKSs are predominant in fungi whereas type III PKSs are found only rarely (Cox 2007; Gallo, Ferrara and Perrone 2013; Hashimoto, Nonaka and Fujii 2014). Genes involved in the synthesis of SMs are frequently located in close proximity to each other, forming so-called secondary metabolite clusters (SMCs) (Keller and Hohn 1997; Brakhage and Schroeckh 2011; Wiemann and Keller 2014). Most of these SMCs contain one biosynthetic core gene that is flanked by transporter proteins, transcription factors and genes encoding tailoring enzymes that modify the SM structure (Keller and Hohn 1997; Keller, Turner and Bennett 2005).

The genomics era has provided new tools to study fungal SMs and their biosynthesis at the whole genome scale (Wiemann and Keller 2014; Medema and Fischbach 2015). The distinctive traits of gene clusters (e.g. gene distance) and the conserved signatures of core genes (e.g. conserved domains) can be exploited to identify putative loci involved in SM production. Moreover, phylogenetic and comparative genomics analyses are very informative as the number of fungal genomes and characterised SM pathways increases. These two approaches are very helpful to identify gene clusters that are involved in the production of SMs that have been characterised in other fungal species and allow subsequent predictions of identical or related compounds that a particular fungal species might produce (Medema , Takano and Breitling 2013; Cairns and Meyer 2017).

The fungal genus *Verticillium* contains nine haploid species plus the allodiploid *Verticillium longisporum* (Inderbitzin *et al*. 2011; Depotter *et al*. 2017). These ten species are phylogenetically subdivided into two clades; Flavexudans and Flavnonexudans (Inderbitzin *et al*. 2011; Shi‐Kunne *et al*. 2018). The Flavnonexudans clade comprises *Verticillium nubilum, Verticillium alfalfae, Verticillium nonalfalfae, Verticillium dahliae* and *V. longisporum*, while the Flavexudans clade comprises *Verticillium albo-atrum, Verticillium isaacii, Verticillium tricorpus, Verticillium klebahnii* and *Verticillium zaregamsianum* (Inderbitzin *et al*. 2011). Among these *Verticillium* spp., *V. dahliae* is the most notorious plant pathogen that is able to cause disease in hundreds of plant species (Fradin and Thomma 2006; Inderbitzin and Subbarao 2014). Furthermore, *V. albo-atrum, V. alfalfae, V. nonalfalfae* and *V. longisporum* are pathogenic, albeit with narrower host ranges (Inderbitzin and Subbarao 2014). Although the remaining species *V. tricorpus, V. zaregamsianum, V. nubilum, V. isaacii* and *V. klebahnii* have incidentally been reported as plant pathogens, they are mostly considered as saprophytes that thrive on dead organic material and their incidental infections should be seen as opportunistic (Ebihara *et al*. 2003; Inderbitzin *et al*. 2011; Gurung *et al*. 2015). Previously, studies of three genes that are involved in SM biosynthesis in *V. dahliae* suggested that SMs may play a role in *V. dahliae* virulence. The deletion mutants of the putative secondary metabolism regulators *VdSge1* (Santhanam and Thomma 2012) and *VdMcm1* (Xiong *et al*. 2016) displayed reduced virulence when compared with the wild type *V. dahliae* strain. Likewise, a reduction in virulence was observed for deletion mutants of the cytochrome P450 monooxygenase *VdCYP1* (Zhang *et al*. 2016), a common tailoring enzyme in SMC production. In this study, we conducted an *in silico* analysis to unravel the potential secondary metabolism of *V. dahliae* by making use of the gapless genome assembly of strain JR2 (Faino *et al*. 2015)

## MATERIALS AND METHODS

### Secondary metabolite cluster prediction, annotation and conservation

Putative SMCs were identified with antiSMASH fungal version 4.0.2 (Weber *et al*. 2015). The predicted borders from antiSMASH were directly used to retrieve all protein sequences contained within the clusters. The Bedtools intersect command (Quinlan and Hall 2010) was used to obtain the file containing the gene locations, followed by gffread from the Cufflinks package (Trapnell *et al*. 2010) to retrieve the protein sequences. Sub-telomeric regions were defined as 300 kb of the chromosomal ends, as similarly used for other filamentous fungi (McDonagh *et al*. 2008; Cairns and Meyer 2017). Genes within the genomic range were counted using BioMart from Ensembl (Kersey *et al*. 2016). A *χ*^2^ test was performed to determine the significance of enrichment.

The conservation of predicted SMCs among *Verticillium* spp. was assessed based on core enzyme conservation, using BLAST + tool protein blast (blastp) (Camacho *et al*. 2009) on predicted protein databases (e-value < 1 × 10^−5^, query coverage > 60% and identity > 50% (Sbaraini *et al*. 2017).

### Phylogenetic analysis

The previously described type I PKSs, NRPSs and PKS-NRPSs enzymes used in this study for phylogenetic analysis were derived from the curated database of UniProt, SwissProt and literatures (Gallo, Ferrara and Perrone 2013; Yu *et al*. 2015). The amino acid alignment was built using MAFFT version 7.205 (Katoh and Standley 2013). We used the G-INS-i strategy, global alignment (–globalpair) and 1000 cycles of iterative refinement (–maxiterate 1000). Aligned sequences were visualised with Aliview version 1.20 (Larsson, 2014) and manually curated by removing non-aligned sequences. Preceding the phylogenetic analysis, the alignments were trimmed to remove poorly aligned regions using TrimAl version 1.4 (Capella-Gutiérrez, Silla-Martínez and Gabaldón 2009). First, all positions in the alignment with gaps in 90% or more of the sequence were removed (-gt 0.1), followed by the automated1 parameter (-automated1). RaxML version 8.1.1 (Stamatakis 2014) was used to construct Maximum-likelihood phylogenetic tree (-f a). The automated protein model selection (-m PROTGAMMAAUTO) was used applying 100 rapid bootstrapping (-#100). The number of seeds for parsimony inferences and rapid bootstrap analysis was set to 12 345 (-p 12 345 -x 12 345, respectively). The output tree (RaxML_bipartitionBranchLabels) was visualised using iTOL webtool version 3.0 (Letunic and Bork 2016).

### Comparative cluster analysis

Protein sequences of described clusters were blasted (BLASTp, E-value cutoff 1e-5, query coverage > 60% and identity > 25%) against the *V. dahliae* strain JR2 protein database. We considered a cluster to be conserved in *V. dahliae* when at least 50% of the queried proteins from previously described clusters were found in *V. dahliae*.

### Gene expression analysis

To obtain RNA-seq data for *V. dahliae* grown in culture medium, strain JR2 was grown for 3 days in potato dextrose broth (PDB) in three biological replicates. To obtain RNA-seq data from *V. dahliae* grown *in planta*, three-week-old *Arabidopsis thaliana* (Col-0) plants were inoculated with strain JR2. After root inoculation, plants were grown in individual pots in a greenhouse under a cycle of 16 h of light and 8 h of darkness, with temperatures maintained between 20 and 22°C during the day and a minimum of 15°C overnight. Three pooled samples (10 plants per sample) of complete flowering stems were used for total RNA extraction. Total RNA was extracted based on TRIzol RNA extraction (Simms, Cizdziel and Chomczynski 1993). cDNA synthesis, library preparation (TruSeq RNA-Seq short insert library) and Illumina sequencing (single-end 50 bp) was performed at the Beijing Genome Institute (BGI, Hong Kong, China). In total, ∼2 Gb and ∼1.5 Gb of filtered reads were obtained for the *V. dahliae* samples grown in culture medium and *in planta*, respectively. RNAseq data were submitted to the SRA database and can be accessed through BioProject accession PRJNA473305.

The RNA sequencing reads were mapped to their previously assembled genomes using the Rsubread package (v1.32.2) in R (Liao *et al*. 2013). The comparative transcriptomic analysis was performed with the package edgeR in R (v3.4.3) (Robinson, McCarthy and Smyth 2010; McCarthy, Chen and Smyth 2012). Genes are considered differentially expressed when *P*-value < 0.05 with a log2-fold-change ≥ 1. *P*-values were corrected for multiple comparisons according to Benjamini and Hochberg (1995).

## RESULTS AND DISCUSSION

### The *V. dahliae* strain JR2 genome contains 25 putative secondary metabolite clusters

**Figure 1.**
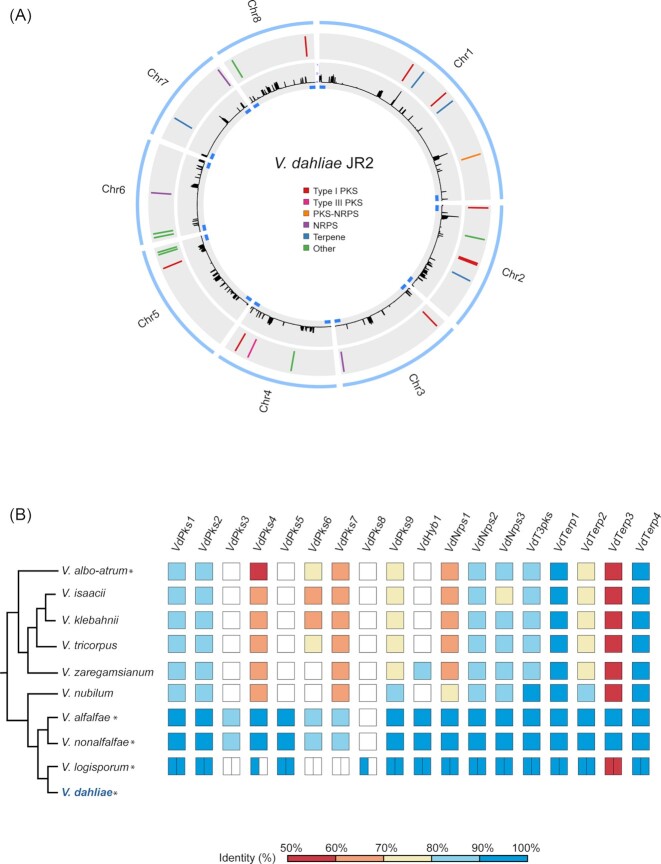
*In silico* predicted *V. dahliae* SMCs. **(A)** Genomic location of *V. dahliae* SMCs. The outer blue lane represents the chromosomes. The middle grey lane shows the relative position of the predicted SMCs on each chromosome. The inner grey lane shows the repeat density in the JR2 genome. The blue rectangles indicate the regions that are defined as sub-telomeric (300 kb from each chromosomal end). **(B)** Conservation of *Verticillium dahliae* core SMC genes throughout the *Verticillium* genus. The colour gradient represents the % identity range of the high scoring. Species described as plant pathogens are indicated with an asterisk. For *V. longisporum*, boxes are divided in two because of its allodiploid nature.

To assess the potential secondary metabolism of *V. dahliae* strain JR2, we mined its genome sequence to predict SMCs using antiSMASH (Weber *et al*. 2015). A total of 25 putative SMCs were predicted, containing a total of 364 genes within their boundaries (Fig. 1 and Table 1). The putative SMCs were classified as nine type I PKSs, one type III PKS, one PKS-NRPS, three NRPSs and four terpenes. Seven clusters were classified as ‘other’, a generic class of SMCs containing core enzymes with unusual domain architecture, also known as non-canonical. We found that each of these SMCs contains one core gene. Disrupted SMCs frequently occur in the genomes of filamentous fungi (Collemare *et al*. 2014). However, none of the clusters identified in *V. dahliae* strain JR2 is evidently disrupted, as all identified clusters have no insertions of transposible elements and include genes encoding tailoring enzymes such as methyltransferases, cytochrome P450 or dehydrogenases (Cacho, Tang and Chooi 2015). Several clusters also comprise transporter and transcription factor encoding genes that might be involved in SM secretion and local gene cluster regulation respectively (Collemare *et al*. 2014) (Table 1). Collectively, these results suggest that all the analysed SMCs of *V. dahliae* strain JR2 are potentially functional.

**Table 1.** Predicted secondary metabolite gene clusters (SMCs) in *V. dahliae* strain JR2

SM class	Predicted key biosynthetic gene	Gene name	Sub-teromeric^5^	Transporter	Transcription factor	Cluster location	No. of genes
T1 PKS^1^	VDAG_JR2_Chr1g1100	VdPks1	NO	YES	YES	1:3 545 717:3 591 771	16
	VDAG_JR2_Chr1g1588	VdPks2	NO	NO	YES	1:5 097 044:5 143 749	15
	VDAG_JR2_Chr2g0045	VdPks3	YES	YES	YES	2:92 065:145 583	18
	VDAG_JR2_Chr2g0695	VdPks4	NO	YES	YES	2:2 119 540:2 167 273	14
	VDAG_JR2_Chr2g0719	VdPks5	NO	YES	NO	2:2 182 651:2 230 558	20
	VDAG_JR2_Chr3g0093	VdPks6	NO	YES	NO	3:316 303:358 028	14
	VDAG_JR2_Chr4g1125	VdPks7	NO	NO	NO	4:3 709 416:3 756 014	17
	VDAG_JR2_Chr5g1015	VdPks8	NO	YES	YES	5:3 384 693:3 432 789	11
	VDAG_JR2_Chr8g1031	VdPks9	YES	YES	YES	8:3 058 903:3 104 627	21
PKS-NRPS^2^	VDAG_JR2_Chr1g23880	VdHyb1	NO	YES	YES	1:7 618 782:7 670 067	20
NRPS^3^	VDAG_JR2_Chr3g1324	VdNrps1	YES	YES	YES	3:4 066 099:4 121 170	19
	VDAG_JR2_Chr6g0600	VdNrps2	NO	YES	YES	6:1 768 180:1 819 114	17
	VDAG_JR2_Chr7g1025	VdNrps3	YES	NO	YES	7:3 179 393:3 227 591	13
T3PKS^4^	VDAG_JR2_Chr4g0955	VdT3pks	NO	YES	YES	4:3 205 819:3 247 084	14
Terpene	VDAG_JR2_Chr1g1245	VdTerp1	NO	NO	NO	1:4 010 610:4 031 875	7
	VDAG_JR2_Chr1g1723	VdTerp2	NO	NO	NO	1:5 472 865:5 493 695	8
	VDAG_JR2_Chr2g0913	VdTerp3	NO	NO	NO	2:2 797 712:2 819 868	10
	VDAG_JR2_Chr7g0295	VdTerp4	NO	NO	YES	7:844 777:866 572	5
Other	VDAG_JR2_Chr2g0388	Other1	NO	YES	YES	2:1 232 486:1 276 127	15
	VDAG_JR2_Chr4g0468	Other2	NO	YES	YES	4:1 587 321:1 632 662	16
	VDAG_JR2_Chr5g1148	Other3	YES	YES	YES	5:3 902 983:3 948 100	14
	VDAG_JR2_Chr5g1176	Other4	YES	YES	YES	5:3 993 259:4 036 240	15
	VDAG_JR2_Chr6g0066	Other5	YES	YES	YES	6:150 534:194 469	14
	VDAG_JR2_Chr6g0109	Other6	YES	YES	NO	6:289 905:335 580	18
	VDAG_JR2_Chr8g0117	Other7	YES	YES	NO	8:289 821:333 602	13

1 T1PKS = type 1 polyketide synthase.

2 NRP-PKS = hybrid polyketide synthase-non-ribosomal peptide synthase.

3 NRPS = non-ribosomal peptide synthase.

4 T3PKS = type 13polyketide synthase.

5 Sub-telomeric clusters were defined as any cluster predicted to reside within 300 kb of a chromosome end.

In several species, SM genes are enriched at chromosomal ends adjacent to the telomeres (Cairns and Meyer 2017; Farman 2007; McDonagh *et al*. 2008). Thus, we assessed whether the SMCs of *V. dahliae* are located in sub-telomeric regions, here defined as within 300 kb from the chromosomal end. We found that 36% of the predicted clusters are in sub-telomeric regions (Fig. 1A and Table 1). As these sub-telomeric regions harbour 13.8% of the total gene repertoire (1606 genes in total) (Table 1), they are significantly enriched (*χ*^2^-test, *P *< 0.0001) in secondary metabolism genes (Fig. 1A).

To check whether the SMCs identified in *V. dahliae* strain JR2 are also present in other *V. dahliae* strains, we assessed the presence/absence of the core SMC enzymes in 22 *V. dahliae* strains (Klosterman *et al*. 2011; Faino *et al*. 2015; Kombrink *et al*. 2017; Depotter *et al*. 2018). Among these 22 strains is the gapless genome assembly of strain VdLs17 (Klosterman *et al*. 2011; Faino *et al*. 2015) and the nearly complete genome assemblies of strains CQ2 and 85S (Depotter *et al*. 2018). The remaining genome assemblies are considerably fragmented, with over 500 contigs for each of the assemblies. Nevertheless, we found that each of the core enzymes is present in all 22 strians, except for VdPks8 which was next to JR2 only found in VdLs17, CQ2 and 85S. However, the absence of VdPks8 from the other strains may be due to the fragmented genome assemblies. Subsequently, we assessed the genome assembles of strains VdLs17, CQ2 and 85S for the presence of complete clusters, revealing that all SMCs identified in *V. dahliae* strain JR2 are also found in the genomes of these three strians, therefore suggesting that SMCs are highly conserved in *V. dahliae* strains.

To examine whether the SMCs identified in *V. dahliae* strain JR2 are also present in other *Verticillium* spp., we queried core enzymes of each cluster using BLAST against the proteomes of previously published *Verticillium* spp. (Depotter *et al*. 2017; Shi‐Kunne *et al*. 2018). In total, 12 SMC core enzymes are present in all *Verticillium* spp., nine of which showed considerable sequnence conservation (>80% sequence identity) (Fig. 1B). The other 15 core enzymes are not present in all species, but display a mosaic presence/absence pattern with presence in at least two other species. Of these, VdPks7 is conserved in all species except for *V. longisporum* (Fig. 1B). VdPks3 is only conserved in the closely related species *V. alfalfae* and *V. nonalfalfae* and VdPks5 is conserved in all pathogenic species except *V. albo-atrum*. Interestingly, the VdPks8 core enzyme was only found in a single copy in the hybrid *V. longisporum* genome, presumably derived from its *V. dahliae* progenitor. Thus, based on the widespread presence of these core genes within the *Verticillium* genus, we predict that most of the SMCs are conserved throughout this genus.

### Phylogenomic analysis of *V. dahliae* secondary metabolite core enzymes

**Figure 2.**
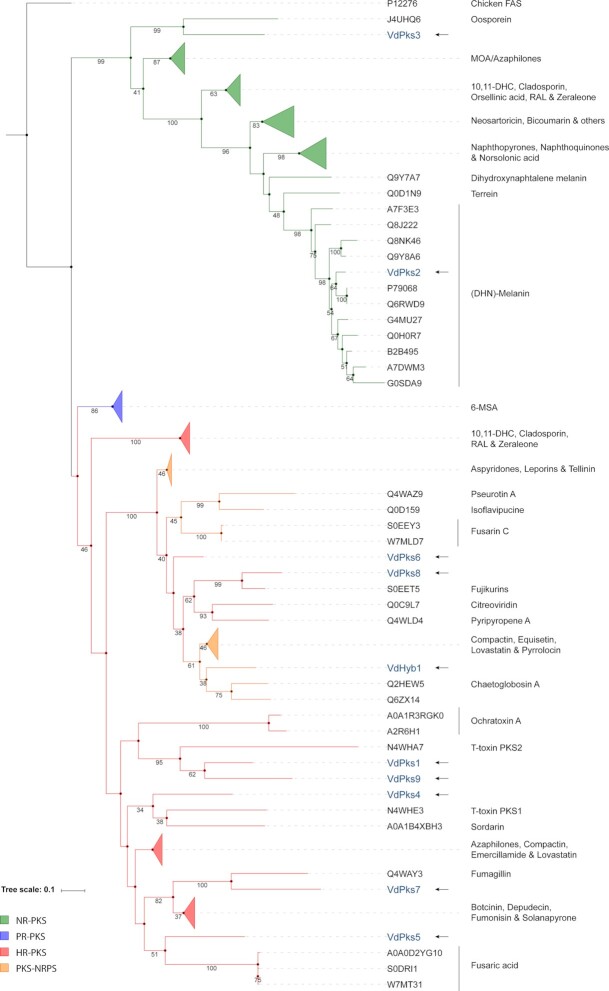
Phylogenetic tree of type I PKS and PKS-NRPS enzymes. KS domains of PKS and PKS-NRPS enzymes were aligned to construct the maximum likelihood tree with 100 bootstrap replicates. The chicken fatty acid synthase (Chicken FAS) sequence was used as outgroup. Only bootstrap values above 30 are shown below the branches. *V*. *dahliae* KS domains are highlighted in blue and indicated with an arrow. Protein codes correspond to Uniprot IDs.

In ascomycetes, type I PKSs, NRPSs and PKS-NRPSs are known to produce most of the SMs that are involved in virulence (Pusztahelyi, Holb and Pócsi 2015). Thus, we focused on identifying putative functions of type I PKSs, NRPSs and PKS-NRPSs in *V. dahliae* with a phylogenomics approach. To this end, we aligned KS domians of *V. dahliae* PKSs to KS domains of functionally described PKSs, and subsequently constructed a phylogenetic tree that comprises three major clades that correspond to the NR-PKSs, PR-PKSs and HR-PKSs, respectively (Fig. 2). The NR-PKS clade contains two predicted *V. dahliae* PKSs, VdPks2 and VdPks3. VdPks2 clusters with PKSs that have been implicated in dyhydroxynaphthalene (DHN)-melanin formation (Tsuji *et al*. 2002; Yu *et al*. 2015). VdPks3 clustered with the Orsellinic acid synthase OpS1, which is involved in production of the toxic metabolite oosporein by the entomopathogenic fungus *Beauveria bassiana* (Feng *et al*. 2015). The HR-PKS clade showed that four out of the seven predicted *V. dahliae* HR-PKSs (VdPks1, VdPks7, VdPks8 and VdPks9) grouped with previously described enzymes. VdPks7 and VdPks8 clustered with the fumagillin synthase from *Aspergillus fumigatus* and fujikurin synthase from *Fusarium fujikuroi*, respectively (Lin *et al*. 2013; von Bargen *et al*. 2015; Niehaus *et al*. 2016). VdPks1 and VdPks9 clustered with the T-toxin synthase PKS2 from *C. heterostrophus* (Inderbitzin, Asvarak and Turgeon 2010). VdPks4 grouped in a clade that contains sdnO and PKS1 synthase, which are involved in the production of Sordarin by *Sordaria araneosa* (Kudo *et al*. 2016), an antifungal agent that inhibits protein synthesis in fungi by stabilising the ribosome/EF2 complex (Justice *et al*. 1998), and in T-toxin production in *C. heterostrophus* (Inderbitzin , Asvarak and Turgeon 2010), respectively. VdPks5 is in a clade that only contains FUB1 fusaric acid synthase orthologs of three *Fusarium* spp. (Brown *et al*. 2015). The remaining *V. dahliae* PKS core enzyme, VdPks6, is not directly grouping adjacent to any previously described enzyme (Fig. 2). Thus, eight of the nine *V. dahliae* PKS core enzymes (VdPks1, VdPks2, VdPks3, VdPks4, VdPks5, VdPks7, VdPks8, VdPks9 and VdHyb1) group with previously characterised enzymes, thereby allowing us to infer their putative function.

**Figure 3.**
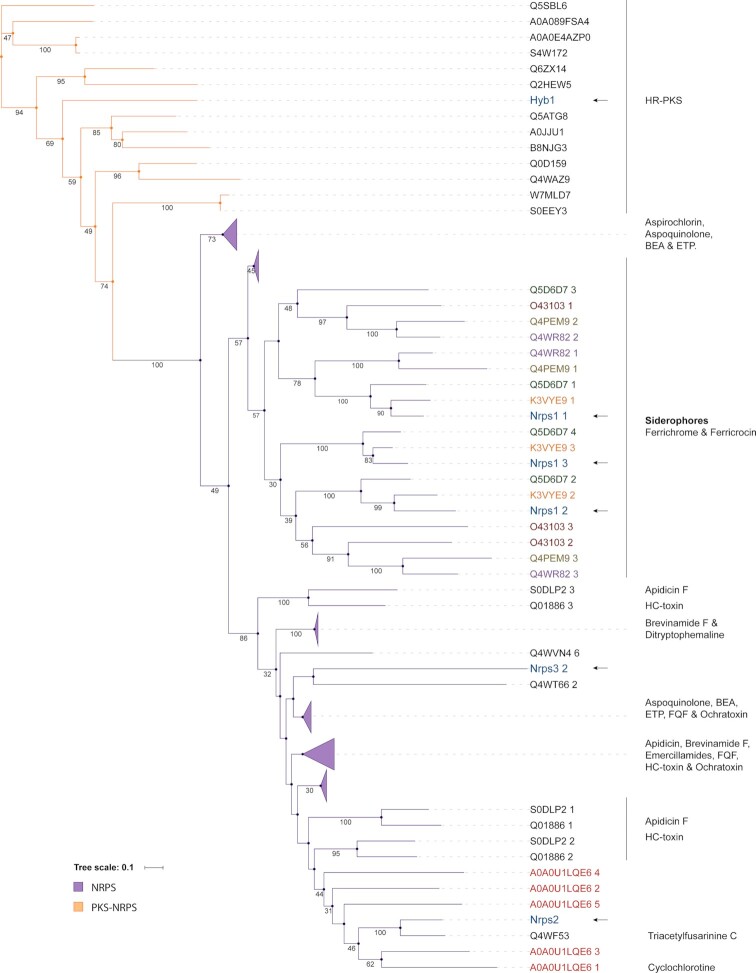
Phylogenetic tree of NRPS and PKS-NRPS enzymes. A-domains of NRPS and PKS-NRPS enzymes were aligned to construct the maximum likelihood tree with 100 bootstrap replicates. Only bootstrap values over 30 are shown below the branches. *V. dahliae* A-domains are highlighted in blue and indicated with an arrow.

Like for PKSs, we similarly performed phylogenomic analysis to get more insight into the putative products of NRPSs. The conserved A-domain sequences of *V. dahliae* NRPS core enzymes were aligned with previously described enzymes of other fungal species to construct a phylogenetic tree. A distinct clade clearly separated NRPSs from the PKS-NRPSs in the phylogenetic tree (Fig. 3). VdNprs1 grouped in a clade with NPS2, which is involved in the synthesis of the intracellular siderophore ferricrocin of *Fusarium pseudograminearum* and *C. heterostrophus* (Tobiasen *et al*. 2007; Sieber *et al*. 2014; Oide *et al*. 2015). VdNprs2 clusters with NRPS4, which is responsible for the synthesis of the extracellular siderophore triacetylfusarinine C (TAFC) by *A. fumigatus* (Schrettl *et al*. 2007). The clade that contains VdNrps3 has low bootstrap values and long branches, indicating considerable divergence of this enzyme (Fig. 3). Thus, only two of the *V. dahliae* NRPS core enzymes (VdNprs1 and VdNprs2) group with previously characterised enzymes.

The fusion of PKS and NRPS domains results in PKS-NRPS enzymes that stand out due to their structural complexity (Boettger and Hertweck 2013). In the genome of *V. dahliae* (strain JR2), only one PKS-NRPS (VdHyb1) was detected. Since it contained a KS domain characteristic for HR-PKSs and an A-domain as commonly observed in NRPSs, we included VdHyb1 in both phylogenetic trees. In the PKS phylogeny (Fig. 2) VdHyb1 was found in the same clade (38%, bootstrap value) as the PKS-NRPSs chaetoglobosin A synthase from *Chaetomium globosum* and the avirulence protein ACE1 from *Magnaporthe grisea* (Collemare *et al*. 2008; Ishiuchi *et al*. 2013). In contrast, VdHyb1 did not cluster with any previously characterised PKS-NRPS in the A-domain phylogenetic tree (Fig. 3).

### Comparative analysis of gene clusters

Based on the phylogenetic analyses, 11 core enzymes (VdPks1, VdPks2, VdPks3, VdPks4, VdPks5, VdPks7, VdPks8, VdPks9, VdNrps1, VdNrps2 and VdHyb1) were identified that cluster with previously characterised enzymes from other fungal species (Figs. 2 and 3). Subsequently, we queried for the other genes besides the core genes from these previously characterised clusters in other fungal species to find homologs in the corresponding *V. dahliae* clusters. However, only the VdPks2, VdPks8, VdNrps1 and VdNrps2 clusters of *V. dahliae* share more homologs (more than 50% of the whole cluster) in addition to the core genes with other fungal species. The remaining clusters contain less than 50% of genes that share homologs with other fungal species. In other fungi, conserved gene clusters of VdPks2, VdPks8, VdNrps1 and VdNrps2 are responsible for the biosynthesis of DHN-melanin, fujikurins, ferricrocin and TAFC, respectively (Tsuji *et al*. 2002; Tobiasen *et al*. 2007; Sieber *et al*. 2014; von Bargen *et al*. 2015; Niehaus *et al*. 2016).

**Figure 4.**
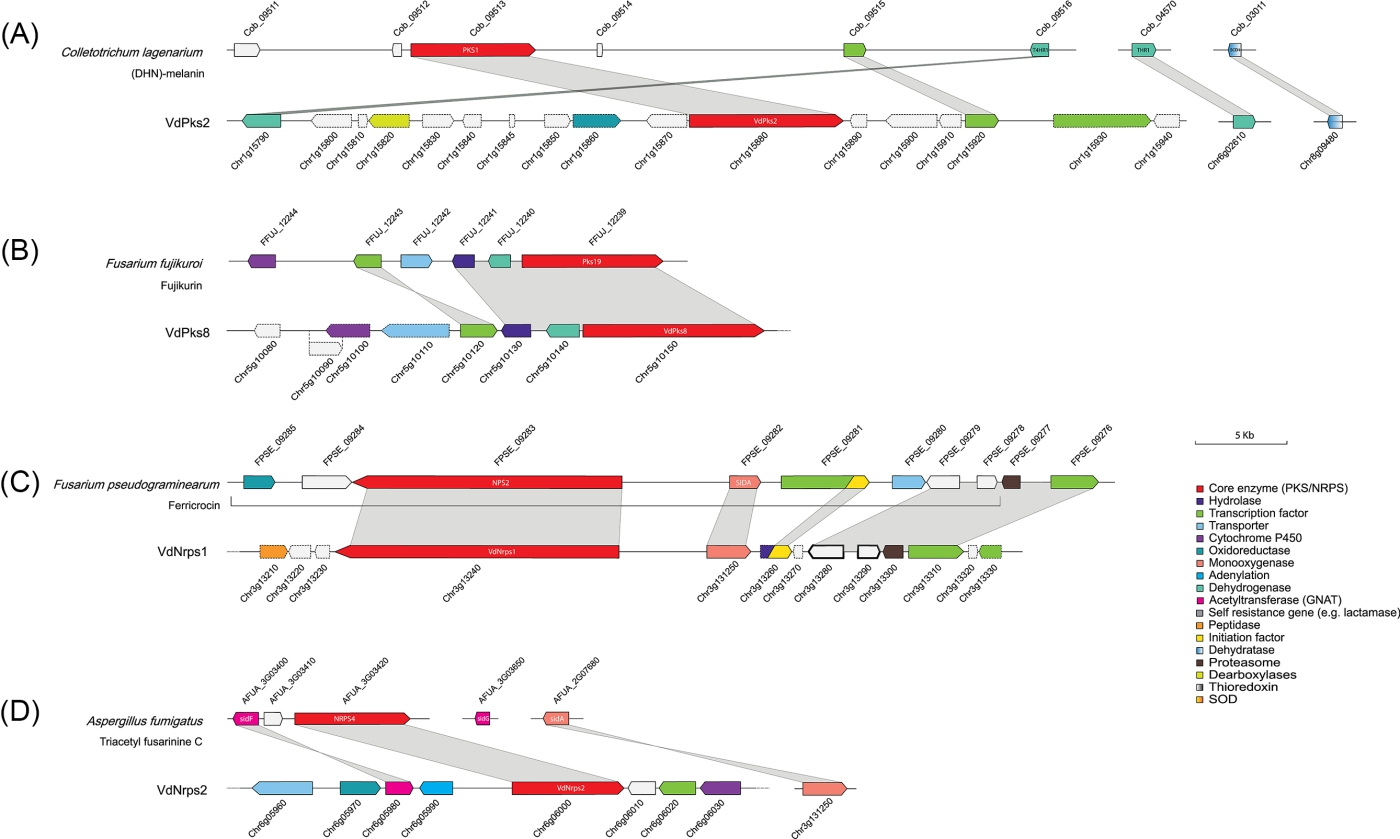
Synteny of conserved SMCs in V. dahliae. V. dahliae putative clusters were compared to previously described clusters. Ensembl gene IDs are shown above or below the genes. **(A)** DHN-melanin, **(B)** Fujikurins, **(C)** Ferricrocin, **(D)** Triacetyl fusarinine.

The gene cluster for DHN-melanin biosynthesis in the fungal pathogen *C. lagenarium* comprises six genes, including three functionally characterised genes encoding PKS *ClPKS1*, reductase *T4HR1* and transcription factor *cmr1*. Moreover, two genes (scytalone dehydratase *SCD1* and *THR1* reductase) residing at another chromosome were identified to be involved in the biosynthesis of DHN-melanin as well (Tsuji *et al*. 2002) (Fig. 4A). We observed amino acid identities of 70%–90% between ClPKS1, T4HR1, SCD1 and THR1 of *C. lagenarium* and their orthologs in *V. dahliae*. The transcription factor CMR1 only shares 55% identity with its counterpart in *C. lagenarium*.

*VdPks8* is an ortholog of the fujikurin synthase gene (*FfuPks19*) of *F. fujikuroi*. The fujikurin cluster contains six genes (von Bargen *et al*. 2015; Niehaus *et al*. 2016), four of which have homologs in the *VdPks8* locus in *V. dahliae*. The homolog of the MFS transporter FFUJ_12 242 of *F. fujikuroi* was found on a different chromosome in *V. dahliae*, and the cytochrome P450 gene in *F. fujikuroi* has no *V. dahliae* homolog (Fig. 4B). Interestingly, the *VdPks8* locus contains two other genes that are annotated as cytochrome P450 and MFS transporter, but these genes were not detected as homologs of the genes in the *FfuPks19* cluster (Fig. 4B).

The biosynthesis of ferricrocin requires two genes that are located at the same locus in the genome of *F. pseudograminearum*, which encode an L-ornithine N5-oxygenase (SIDA) and an NRPS (FpNRPS2) (Tobiasen *et al*. 2007; Sieber *et al*. 2014). Similarly, in *V. dahliae* homologs of L-ornithine N5-oxygenase (SIDA) and NRPS (FpNRPS2) genes are located next to each other. Moreover, homologs of a proteasome subunit, a transcription factor and two uncharacterised genes in the same cluster of *F. pseudograminearum* were found in *V. dahliae*. In addition, genes encoding an MFS transporter and an oxireductase in *F. pseudograminearum* have no homologs in *V. dahliae* (Fig. 4C).

VdNrps2 is an ortholog of the extracellular siderophore TFAC synthase gene *NRPS4* in *A. fumigatus*, which belongs to a cluster of two genes (*NRPS4* and sidF) (Schrettl *et al*. 2007). Another two extracellular siderophore TFAC synthase genes (*sidG and sidA*) are located at another chromosome (Schrettl *et al*. 2007). Except for *sidG*, all described genes have homologs in *V. dahliae* (Fig. 4D).

### Gene cluster expression analyses

**Figure 5.**
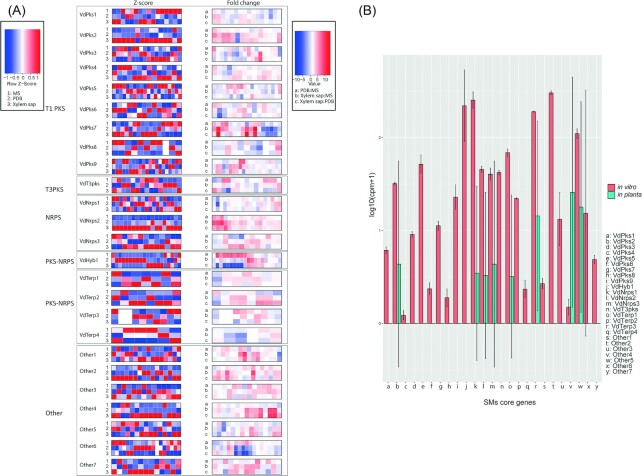
SMC gene expression analyses in *V. dahliae* strain JR2. **(A)** Relative expression (Z-scores, calculated based on read counts) of SMC genes upon fungal growth *in vitro* (left panel) and pair-wise comparison of SMC genes with differential expression *in vitro* (right panel). **(B)** Pair-wise comparison of core SMC genes with differential expression *in vitro* and *in planta*. Gene expression is depicted upon growth in potato dextrose broth and upon *A. thaliana* colonisation, respectively. Bars represent the mean gene expression with standard deviation. The significance of difference in gene expression was calculated using *t*-tests relative to a threshold (TREAT) of log2-fold-change ≥ 1 (McCarthy and Smyth 2009).

To identify differentially expressed SMCs, we queried RNA-seq data sets from *V. dahliae* grown *in vitro* in PDB, Murashige and Skoog (MS) medium and xylem sap that was harvested from healthy tomato plants. In order for a gene cluster to qualify as differentially expressed, genes within the clusters must show similar expression patterns and the expression must differ between conditions. However, we found that the majority of gene clusters do not show such expression patterns, meaning that the genes within a cluster do not appear to be co-regulated. Only the clusters VdPks2, VdNrps2, VdNrps3, VdTerp2 and Other4 show co-regulated expression patterns, since the majority of genes within those clusters (>75%) have similar relative expression levels in at least two conditions (Fig. 5A). Furthermore, these five clusters show differential expression (>2 fold) under those two conditions. Interestingly, all five clusters show higher relative expression in xylem sap when compared with PDB and MS medium.

To assess the potential of SM clusters to play a role in pathogenicity, we assessed transcriptome (RNA-seq) data of *V. dahliae* during colonisation of *A. thaliana* plants. We found that the majority of SMC genes are not expressed *in planta*, although eight core SMC genes are expressed under those conditions (Fig. 5B). We therefore compared the relative expression levels of each core gene *in planta* and *in vitro* (PDB), whereas the *Other4* core gene was found to be induced *in planta*, the core genes of *VdPks2*, *VdNrp1*, *VdNrps2 VdNrps3, VdTerp1, VdTerp3* and *Other5* were repressed when compared with the expression in *in vitro*-cultured mycelium (Fig. 5B). This finding may suggest that *Other4* plays a role during colonisation of *A. thaliana*. However, it cannot be excluded that the six core genes that are down-regulated *in planta* still play a role during host colonisation as well since residual expression remains.

## CONCLUSIONS

In this study, we have used an *in silico* approach to identify 25 putative SMCs in the genome of *V. dahliae* strain JR2, all of which appear complete and thus potentially functional. Our predictions state that two putative siderophores, ferricrocin and TAFC, DHN-melanin and fujikurin compounds may belong to the active SM repertoire of *V. dahliae*.
